# Using *In Situ* Polymerization to Increase Puncture Resistance and Induce Reversible Formability in Silk Membranes

**DOI:** 10.3390/ma13102252

**Published:** 2020-05-14

**Authors:** Nicholas S. Emonson, Daniel J. Eyckens, Benjamin J. Allardyce, Andreas Hendlmeier, Melissa K. Stanfield, Lachlan C. Soulsby, Filip Stojcevski, Luke C. Henderson

**Affiliations:** Carbon Nexus, Institute for Frontier Materials, Deakin University, Waurn Ponds, VIC 3216, Australia; nemonson@deakin.edu.au (N.S.E.); dan.eyckens@deakin.edu.au (D.J.E.); ben.allardyce@deakin.edu.au (B.J.A.); ajhendlm@deakin.edu.au (A.H.); mstanfie@deakin.edu.au (M.K.S.); l.soulsby@deakin.edu.au (L.C.S.)

**Keywords:** aryldiazonium, surface modification, surface chemistry, silk membrane

## Abstract

Silk fibroin is an excellent biopolymer for application in a variety of areas, such as textiles, medicine, composites and as a novel material for additive manufacturing. In this work, silk membranes were surface modified by *in situ* polymerization of aqueous acrylic acid, initiated by the reduction of various aryldiazonium salts with vitamin C. Treatment times of 20 min gave membranes which possessed increased tensile strength, tensile modulus, and showed significant increased resistance to needle puncture (+131%), relative to ‘untreated’ standards. Most interestingly, the treated silk membranes were able to be reversibly formed into various shapes via the hydration and plasticizing of the surface bound poly(acrylic acid), by simply steaming the modified membranes. These membranes and their unique properties have potential applications in advanced textiles, and as medical materials.

## 1. Introduction

Silk as a material shows incredible diversity in applications and has been used for millennia in areas such as textiles; it now has modern applications in medicine, and has FDA approval [1]. Taking native silk and removing sericin, a gummy material used to glue the fibres together, reveals the fibroin core—a biopolymer consisting of mostly alanine and glycine with minor constituents of serine and tyrosine. The fibroin can be dissolved and then regenerated in a large variety of forms, and processing conditions can be altered to tune fibroin’s secondary structure, influencing the physical properties of the regenerated material [2,3,4,5,6]. Overall, the properties of silk fibroin are impressive, though they are still lacking in certain areas when used in films or membranes. Without the use of additives/plasticisers, the membranes tend to be very brittle, exhibiting brittle fracture at elongations typically less than 5%. The use of hydrogen bonding additives such as glycerol has addressed the brittle nature of the films but at the expense of mechanical properties, and they still possess a narrow window of elastic deformation [7].

The use of silk as a material in composites is of high interest given its unique properties, biocompatibility, and ability to be processed into different forms [8,9,10,11,12]. The effective use of silk in a structural or functional material would open up huge possibilities in the reuse of silk textile waste instead of sending this to landfill [13]. Additional waste streams can arise from unusable floss surrounding the silk cocoon and spinning waste, among others. The ability to surface modify silk fibroin at a chemical level proves challenging as the amino acids present in the vast majority of the backbone (alanine, glycine) are not reactive under benign conditions. Similarly, serine and tyrosine, which possess alcohol and phenol units, respectively, are present in relatively low (5–15%) abundance, and due to the role of these amino acids in hydrogen bonding, it may mean that these functional groups are not readily available for modification. Nevertheless, there have been recent reports of modifying the tyrosine residue in the solid membrane via the use of aryldiazonium salts [14,15,16] and elaboration of this modified material using copper-azide-alkyne cycloaddition (‘Click’) chemistry to install a plethora of surface chemistries [17]. Alternatively, this approach can be carried out before solution casting to give a homogenously modified material (vs. periphery-only modified), which was used for ligating polysaccharides [18]. Recently, another approach examining the modification of serine was also reported when examining the adhesive properties of silk fibroin to leather surfaces [19].

**Figure 1:**
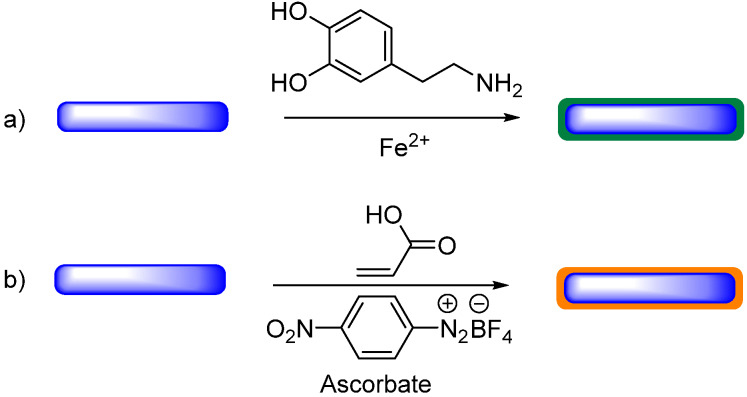
(**a**) An approach for polymer growth on silk membranes using polydopamine [28]; (**b**) the approach presented herein.

Another method is to use the silk fibroin structure as a template for the growth or deposition of materials onto its surface using established methodologies such as the oxidative polymerization of polydopamine [20] (Figure 1), deposition of graphene and graphene oxide [21], sol-gel processes [22], among other common materials [23,24,25,26,27]. These approaches have demonstrated success for the manipulation of such characteristics as super hydrophobicity, increasing hydrophilicity, and increasing tensile strength, to name a few.

**Figure 2:**
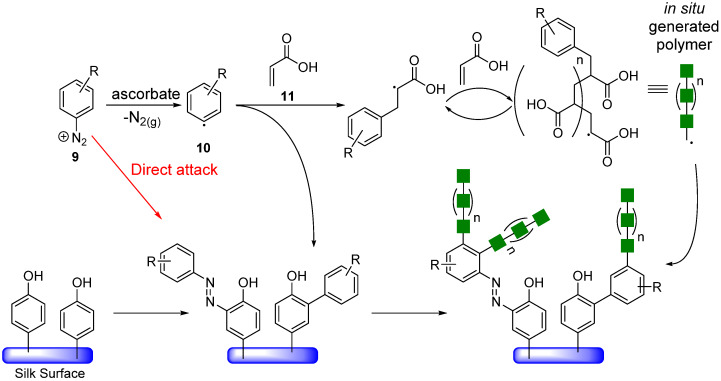
An overview of the chemical processes used in this study to modify the surface of silk fibroin membranes with poly(acrylic acid) 11.

In this work, we report the adaptation of an *in situ* polymerization procedure, first reported in 2006 by Deniau et al. [29], to silk membranes. This process is an elegant mixture of ‘graft to’ and ‘graft from’ techniques, which uses the reduction of aryldiazonium salts (typically 4-nitrobenzene diazonium tetrafluoroborate 1) to both initiate the polymerization of acrylate-derived monomers and enrich the surface (of the material to be modified) with aryl rings. These aryl rings serve as a ‘priming layer’ for the radicals of the polymer chains to attach to (Figure 2). This process is applicable to a wide array of conductive materials (metals, carbons, etc.) and was recently applied to carbon fibers to great effect [30]. The monomer of interest in this report is acrylic acid, which is generated as a by-product of ethylene and gasoline production.

In the original reports by Deniau et al. [29,31,32], a reductive electrochemical potential was used to reduce **9** to the corresponding aryl radical, due to the reliable and irreversible single electron reduction of aryldiazonium species. Since then, this reduction has subsequently been shown to be affected by hypophosphorus acid, metallic iron, and selected other chemical reductants [32]. Given the non-conductive nature of silk fibroin, the development of a chemical functionalization protocol which is both effective in grafting a polymer solution to the surface of the membranes while not compromising the structural integrity of the biopolymer is of utmost importance.

This paper reports a rapid and procedurally simple method of surface-initiated polymerisation on silk fibroin membranes. These membranes are characterised using water contact angle (WCA), Fourier Transform Infrared Spectroscopy (FT-IR), atomic force microscopy (AFM), and evaluated for their puncture resistance. The result of the surface bound polymer is a significant increase in the puncture resistance and the ability to reversibly form these membranes after exposing them to steam.

## 2. Experimental

### 2.1. Materials

The silk membranes used in this study were fabricated according to previously published procedures [7]. Typically, this a membrane cast with in a ratio of 40 mg of glycerol/100 mg of silk.

### 2.2. Surface Modification of Silk Fibroin Films

A piece of 40% glycerol (Sigma-Aldrich, St Louis, MO, USA) casted silk membrane (designated untreated) (1 cm^2^) was dipped in ascorbic acid (Sigma-Aldrich, St Louis, MO, USA) (0.02 M) and air dried for 12–24 h. A vial was then filled with sulfuric acid (Sigma-Aldrich, St Louis, MO, USA) (10 mL, 0.01 M) and diazonium salt (2 mM) prepared according to literature procedure [30]. This solution was stirred for 10 min to allow the diazonium salt to dissolve before adding acrylic acid (Sigma-Aldrich, St Louis, MO, USA) (480 μL). If it had not dissolved, it was sonicated for 10 min before adding the acrylic acid. A piece of ascorbic acid-coated silk membrane was then placed in the vial and stirred for 20 min at room temperature. The membranes were removed, placed in clean vials and washed with water and ethanol (10 mL) for 5 min each. The silks were air dried for 12–24 h and stored in vials for analysis.

Note that the control sample was an untreated membrane which was subjected to the above reaction conditions, but in the absence of the aryldizaonium salt.

### 2.3. Atomic Force Microscopy

The surface roughness of the silk samples was determined using atomic force microscopy (AFM). Two AFM instruments were used to obtain the surface topography of the samples, a Bruker Dimension SPM 3000 microscope (Billerica, MA, USA) and a Bruker Multimode 8 AFM (Billerica, MA, USA). A silicon nitride pyramid probe was used for both of the microscopes and the spring constant of the cantilever was 0.12 N/m. The silk membranes were mounted onto glass or magnetic slides to ensure flatness and stability during testing. Contact mode mapping was then used across the silk surfaces at a scan rate of 0.5 μm/min to obtain five 5 μm × 5 μm images of each sample. The images were then imported to NanoScope Analysis 1.4 and the arithmetic roughness average (*R_a_*) was calculated using Equation (1) shown below, where *Z*(*x*) is the depth of the peaks and troughs on the silk surface and *L* is the length of the scan.

(1) $$R_{a}=\frac{1}{L}\overset{L}{\underset{0}{\int }}\left|Z\left(x\right)\right|dx$$

### 2.4. Water Contact Angle

For contact angle testing, the silk samples were fixed to glass slides using double-sided tape, ensuring that the surface of the silk was flat. Using a 3 mL syringe a standard drop of 4 μL was used to apply water droplets to each silk sample. The contact angle of the drops with respect to the flat surface was then measured each second for 7 s (allowing 1 s to equilibrate, total test 8 s) using Attension Theta Software (ATA Scientific, Taren Point, Australia) and a micro-lens camera. Each sample was tested using deionised water.

### 2.5. Needle Penetration Tests

Using a Terumo Needle rig (Terumo, Shibyua TYO, Japan) (20G’ 1 ½ (0.90 mm, 38 mm)). Square sections of silk (10 mm × 10 mm) were mounted into a specially designed metal puncture rig (see Supplementary Materials). For each silk configuration, 5 puncture tests were conducted. The needle head was moved at a displacement rate of 0.5 mm/min using a 10 kN load cell with a load sensitivity of 0.2 N. The load vs. displacement curves were analysed with the maximum load required to cause puncture being documented as well as the extension at which this load occurred. We presented these data in Newtons (N), as the area over which the force is distributed cannot accurately be measured as the needle tip is tapered. Therefore, we have assumed that the needle tips are consistent in their shape, and thus the results can be compared internally.

### 2.6. Membrane Malleability Procedure

A 10 mm × 50 mm piece of silk membrane was functionalised (via the method described in Section 2.2). After the pieces of silk were dried for 24 h, they were heated at 96 °C in a closed, moist environment (created using two beakers, one filled with water inside the other enclosed by a foil lid) for 10 min. After this, the lid was slightly opened so as to release as little heat as possible and the silk membranes were placed between two ‘L’ shaped metal brackets and heated for a further two minutes. The membranes were then removed from the beaker and allowed to cool at room temperature for 5 min before removing them from the metal brackets. After cooling for 5 min, the silk membrane was returned to the beaker and reheated at 96 °C. Following this, the silk was removed, cooled and stored for later use.

### 2.7. Statistical Analysis

Data were compared using a two-tailed t-test and equal variance was assumed, ‘*p* values’ less than 0.05 were considered statistically significant.

## 3. Results and Discussion

### 3.1. Optimisation Surface Modification Procedure

Despite the excellent utility and user friendliness of this polymer grafting process, it has seen limited uptake by researchers for any surface in preference for controlled living polymerizations [33]. This is presumably due to the increased control (using the latter method) over polymer molecular weight dispersity and the ability to elaborate the surface grafted materials into di- or multi-block polymers. The increased synthetic fidelity that is provided by RAFT or ATRP often comes at the expense of time and multiple material preparation steps, e.g., ligation of a radical transfer unit (xanthate or α-bromocarbonyl species). In this instance though, an uncontrolled and operationally simple procedure would be preferred to allow for broad applications and to ensure that these materials would not become prohibitively expensive.

**Scheme 1:**
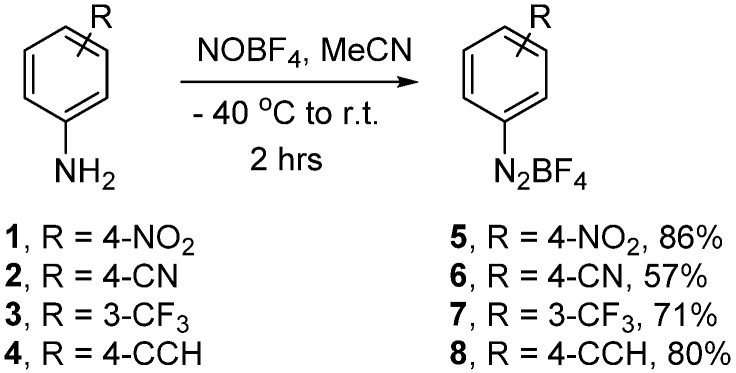
Synthesis of aryldiazonium salts using NOBF_4_.

To this end, there has been only limited examination of which aryldiazonium salts are best suited to this process, and presumably, this will vary on a case-by-case basis depending on the intended acrylic monomer, reaction media, and the nature of the surface to be modified. As such, we qualitatively examined a small suite of aryldiazonium salts **5**–**8** to see which one(s) would perform optimally for this application. These were synthesised according to previously reported procedures [7], and were all synthesised in moderate to excellent yield (Scheme 1).

Given the nature of silk and its potential use in biomedical applications, ascorbic acid was considered to be the most suitable reductant for the aryldiazonium species, as it is biocompatible, made at large scale (making it conducive to scale up), inexpensive, and is easy to handle. The introduction of the ascorbic acid posed a slight problem initially, as placing the silk membrane within the grafting solution (Figure 2, acrylic acid, 5, 0.01 M H_2_SO_4_) then adding the reductant caused the polymerization to initiate at the top of the solution and not near the surface of the silk membrane. The result of this was the deposition of the polymer onto the silk membrane, rather than the growth of the polymer from the surface, potentially leading to issues with polymer–silk adherence at the interface.

To obviate this problem, the silk membranes were pre-impregnated with ascorbic acid by dropping a dilute solution (10 mM) onto the membrane and allowing it to evaporate upon standing, the purpose being that as the membrane is added to the grafting solution, the ascorbic acid is leached from the membrane, immediately reducing any aryldiazonium salt in local proximity to the silk surface (thus mimicking the electrochemical reduction), causing both surface grafting and polymerization initiation simultaneously. In the interest of practicality, a membrane submersion time limit of 20 min was chosen.

Upon addition of these impregnated membranes to the grafting solution, containing aryldiazonium salt **9** (Scheme 1), an immediate colour change was apparent on the silk surface (from clear transparent to orange (see SI for spectrographic data). This is consistent with the introduction of biaryldiazo species (Figure 2, in red) and is unavoidable when using aryldiazonium salts (generic structure 9), as has been investigated previously [29]. Concomitant generation of the aryl radical 10 can either react with exposed phenol units present within the silk due to tyrosine residues, generating a C-C biaryl bond, or diffuse into solution and react with the acrylic acid monomer to initiate the polymerization process. These polymers can then diffuse back to the silk solution and react with the aromatically enriched silk membrane surface, encasing the membrane in a poly(acrylic acid) film.

This process was repeated for aryldiazonium salts **1**–**4**, and similar colour changes were noted over the 20 min reaction period, again suggesting that the biaryldiazo species had been formed on the silk membrane. Interestingly, after the removal of the treated membranes from the grafting solution, and washing with water, the silk membranes were noticeably more rigid and less fragile than the starting material. This was most obvious when the membranes had to be cut into strips for analysis using a freshly opened scalpel. The treated membranes required multiple cuts to successfully cleave the film, while the untreated silk films are much more susceptible to tearing and are easy to cut.

### 3.2. Physical Characterisation of the Treated Membranes

Analysis of the modified films using IR was not definitive as the sample was saturated with the carbonyl stretches of the underlying protein scaffold (see SI). It was noted that the shape of the amide I and amide II peaks, which correspond to the silk structure, did change after treatment, suggesting the influence of another absorbance in this region, such as the carbonyl of the poly(acrylic acid).

**Figure 3:**
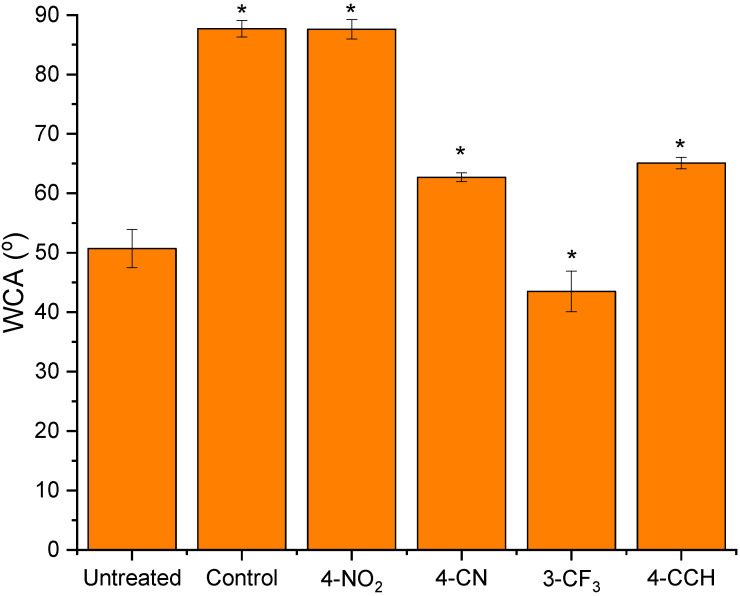
Water contact angles (WCA) for untreated, control, and functionalised membranes used in this study. Errors are standard deviation, * denotes statistically significant difference to untreated sample, *p* < 0.05.

**Figure 4:**
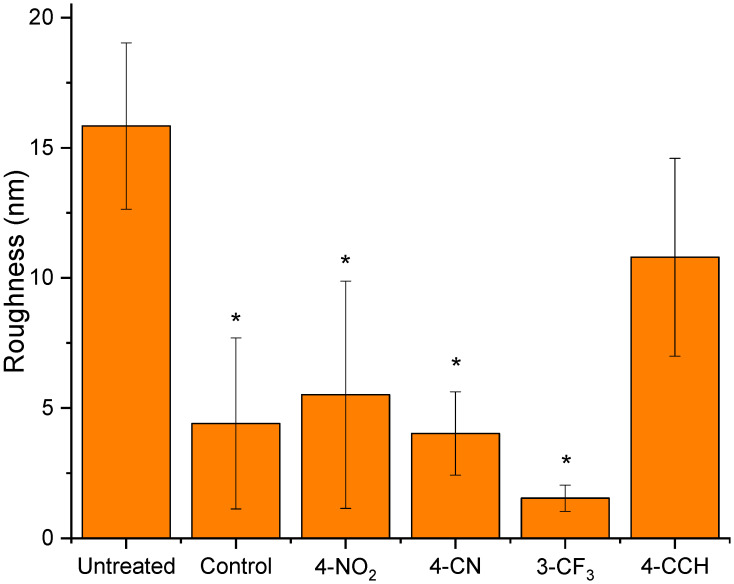
Roughness of modified silk (*R_a_*) membranes determined by atomic force microscopy (AFM). Errors are standard deviation, * denotes statistically significant difference to untreated sample, *p* < 0.05.

Nevertheless, another method of determining the presence of the poly(acrylic acid) on the surface of the silk films was the use of water contact angle (WCA). The presence of such a polar and hydrophilic polymer on the surface of the silk was thought to reduce the WCA substantially. Therefore, taking the untreated silk membranes, which have been cast with a glycerol plasticiser, and measuring the WCA, gave an angle of 50.7° (Figure 3). We used this as our reference point, as these are the membranes which are currently of clinical interest, and as this measurement was used as a broadly qualitative comparison. Also, subtle effects such as glycerol leaching into the water droplet and dynamic changes at the interface were ignored and considered negligible. Therefore, the WCA is provided as the average of seven measurements and is presented as a global value for the sample. In the interest of thoroughness, and to ensure that the reduction of the aryl diazonium salt was responsible for the formation of the polymeric coating, a trial was conducted where the ascorbic acid-impregnated films were immersed in the grafting solution in the absence of any aryldiazonium salt (this sample is designated as ‘control’). The resulting film demonstrated an increased WCA (87.7°, Figure 3), increased by 37° from the untreated sample. This sample was not accompanied by the usual colour change, again, consistent with the absence of aryldiazonium salt and thus no formation of the biaryldiazo species. The increased contact angle is presumably due to either low levels of surface grafting from native amino acid side chains (e.g., lysine, tyrosine, serine, etc.) or through via the leaching of glycerol from the membrane. Interestingly, the use of 4-nitrobenzenediazonium tetrafluoroborate (Figure 3) as the initiator showed an almost identical WCA (87.6°, Figure 4) to the control.

In this instance, similar WCA values from the presence of nitrophenyl groups at the surface of a material were observed by our group previously [34]. This is likely assisted by the diazonium salt reacting extensively via the direct attack pathway (Figure 2, in red). This reaction would be encouraged in this instance as the very strong electron withdrawing nature of the NO_2_ group would increase the electrophilicity of the diazonium moiety. The film was observed to turn the typical red/orange colour, as is consistent with the formation of the –N=N– group, and similar increases in WCA have been observed for multi-layers of nitrophenyl aromatic surfaces in recent reports (see SI for absorbance spectra) [35]. Nevertheless, carrying out this process with the remaining aryldiazonium salts (Figure 3) gave promising results. The polymerization mediated by 3-(trifluoromethyl)benzenediazonium salt **7** gave a very sharp decrease in WCA (43.5°, Figure 3), while the membranes modified using the 4-cyano and 4-ethynylbenzene diazonium salts **5** and **8**, respectively, gave similar WCA values of 62.7° and 65.1°, respectively. Also, for each of the modified membranes, the tactile nature of the membranes was noticeably changed, becoming more brittle. Considering the spread of values when examining the WCA, there is the possibility that the polymeric grafting is not homogeneous, and erroneous results are being obtained depending on the location of the drop on the film. This was indeed the case with the membranes modified using the 4-ethynylbenzenediazonium salt which were noticeably more rigid at the periphery of the membranes while the middle remained lighter in colour and more ductile to the touch than samples modified using either the 4-cyanobenezenediazonium or 3-(trifluoromethyl) benzenediazonium salts **6** and **7**, respectively.

Therefore, the samples were then analysed using AFM to gauge comparative sample roughness and film homogeneity (Figure 4). All samples were analysed in their dry state, under ambient conditions. Using this as a guide, the untreated sample showed an undulating surface (*R_a_* 15.8 ± 3.1 nm), most likely due to the presence of glycerol in the membrane causing localised plasticisation via swelling. This observation is consistent with the control sample, which would have the glycerol leached from the membrane causing it to become much smoother (*R_a_* 4.4 ± 3.8 nm). The use of aryldiazonium salts gave surfaces with roughness values much closer to that of the control sample, with the membrane treated with 4-nitrophenyl tetrafluoroborate giving a surface topologically similar to the control (*R_a_* 5.5 ± 4.4 nm).

This, in combination with the WCA above, would suggest that both the membranes using the 4-cyano and 3-(trifluoromethyl)benzenediazonium salts **6** and **7** gave homogenous films of poly(acrylic acid) onto the silk membrane (*R_a_* 4.0 ± 1.6 and 1.5 ± 0.5 nm, respectively). Using 4-ethynylbenzenediazonium salt **8** gave a roughness similar to the untreated membrane (10.8 ± 3.8 nm), and is consistent with the proposed inhomogeneous grafting of the polymer to the surface of the silk.

Taken together, these analyses would suggest that the best aryldiazonium salts used to graft poly(acrylic acid) to the silk fibroin membrane in this study bear the 4-nitro, 4-cyano, or 3-trifluoromethyl substituents on the aryl ring. The reason for this is presumably to do with the electronics on the aromatic ring and the relative stability of the aryl radical resulting from the *in situ* reduction of the diazonium moiety. Similarly, the diazonium salt bearing the 4-ethynyl group seems to give the desired grafting result despite appearing inconsistent and inhomogeneous across the surface of the silk membrane. Nevertheless, with these data in hand, our attention turned to physically characterising these surface-modified membranes.

### 3.3. Physical Characterisation of Treated Silk Membranes

#### Needle Puncture Test

While preparing the modified silk membranes for various tests (e.g., cutting into strips), the treated membranes were resistant to being cut with a scalpel. This is highly unusual for silk membranes, which are very easily cut and manipulated. Therefore, we were interested in evaluating these membranes for their ability to resist puncture with a syringe. Using a custom-made rig (see SI), we determined the maximum puncture load and deformation at puncture for the treated membranes. It should be noted that all membranes were stored under identical conditions, and thus variations in properties due to moisture absorption or loss were considered to be negligible.

**Figure 5:**
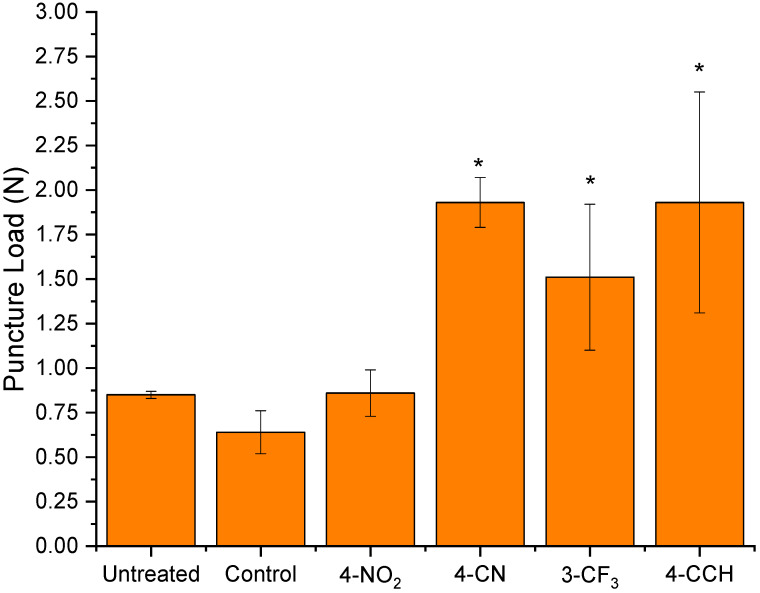
Puncture load (N) for all treated samples, including untreated and control samples. Errors are standard deviation, * denotes statistically significant difference to untreated sample, *p* < 0.05.

**Figure 6:**
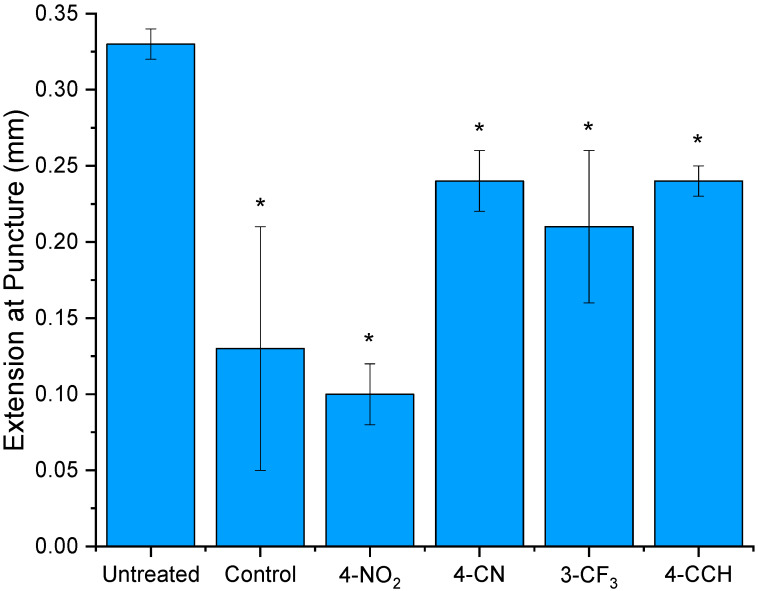
Extension at puncture for all treated samples, including untreated and control samples. Errors are standard deviation, * denotes statistically significant difference to untreated sample, *p* < 0.05.

Testing the untreated membranes displayed a moderate resistance to puncture with a maximum puncture load of 0.85 N (Figure 5), presumably derived from the ability of the plasticised membrane to deform under applied load, in this case 0.33 mm. The removal of the plasticiser by soaking the membranes in the grafting solution (in the absence of diazonium initiator) reinforced this observation as both the puncture load (0.64 N) (Figure 5) and distortional capability (0.15 mm) were reduced (Figure 6). Again, as is consistent with the rest of the observations, the membranes treated in the presence of the 4-nitrobenzenediazonium salt displayed properties very similar to those of the control sample (Figure 5). The only difference was a slightly reduced ability to deform under stress, which may be linked to the formation of biaryldiazo species on the surface.

Interestingly, the remaining samples showed a significant increase in resistance to needle puncture. This was highlighted by both the 4-cyano and 4-ethynylbenzenediazonium treated samples. Figure 5 shows a 131% and 127% improvement in puncture load, respectively. Similarly, the 3-(trifluoromethyl)benzenediazonium treated membranes improved puncture load by 77% (Figure 5). In each of these cases, the distortion of the membranes was improved over that of the control by ~0.1 mm, though had not reached that of the fully plasticised untreated samples (Figure 6). The origin of the significant increase in flexural strength due to the surface treatment is unknown, although it is possibly due to several concurrent effects. The aqueous bath used for grafting likely results in the leaching of glycerol from the membrane, which is a known plasticiser. Additionally, the lamination of the membrane in poly(acrylic acid), effectively making a composite material, may take advantage of the silk’s inherent strength complemented by the typical ductility inherent to thermoplastic polymers.

While these properties and increases in flexural strength were impressive, it is unlikely that any of these materials will be used in an environment completely absent of water and water vapour.

**Figure 7:**
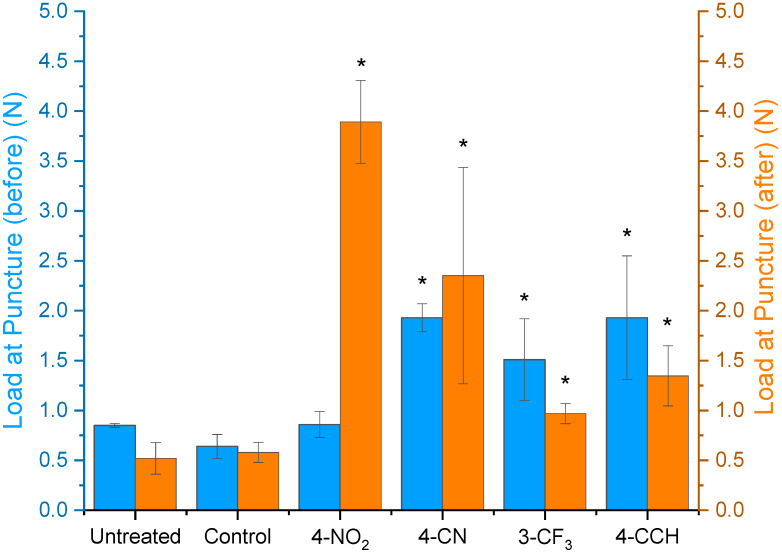
Changes in puncture load (left) and extension at puncture (right) for all treated samples including untreated and control samples, after being rehydrated in a steam bath. Errors are standard deviation, * denotes statistically significant difference to untreated sample, *p* < 0.05.

Therefore, we considered it pertinent to consider the effect of hydrating the bound polymer and determining the physical properties (Figure 7). This was carried out by placing the functionalised membranes in a steam bath and allowing the water vapour to infiltrate the surface-tethered polymer. This should be sufficient as the parent polymer, poly(acrylic acid), is used in water absorption applications and is a known desiccant.

**Figure 8:**
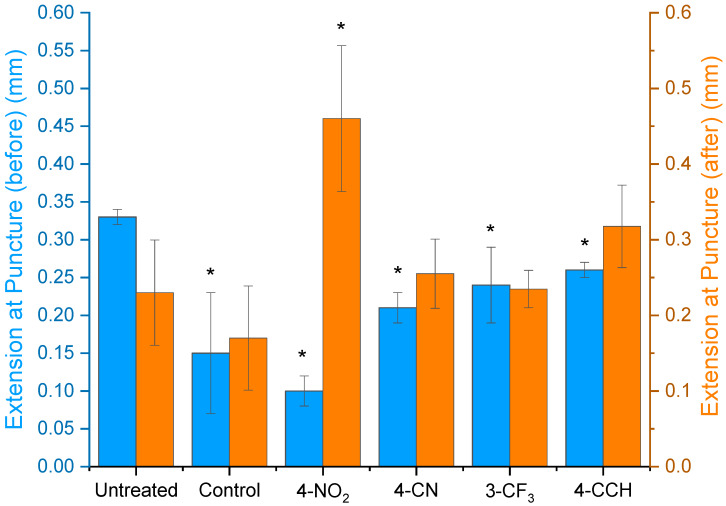
Extension at puncture for all treated samples including untreated and control samples, after being rehydrated in a steam bath. Errors are standard deviation, * denotes statistically significant difference to untreated sample, *p* < 0.05.

The untreated samples gave a slight decrease in both the amount of load tolerated before puncture, before and after steam treatment (0.85 vs. 0.52 N, respectively) and elongation of the material before puncture (0.33 vs. 0.23 mm, respectively, Figure 8). This weakening and increasingly brittle nature of the film is likely due to the leaching of glycerol from the membrane when exposed to a hot and humid environment. The control sample had already been immersed in an aqueous solution, presumably leaching the glycerol from the film prior to this treatment. This correlated with a negligible change in load before puncture before treatment (0.64 N) compared to after (0.58 N) (Figure 7), and elongation increased slightly (0.11 vs. 0.17 mm) (Figure 8).

Interestingly, the exposure of films functionalised using 4-nitrobenzenediazonium salt **3** resulted in significant increases in both the tolerance of load and extension compared to the dehydrated versions. With respect to load tolerance, an increase of 355% (0.86 N vs. 3.89 N, before and after, respectively) was observed, and a similar increase of 351% was noted for elongation at puncture (0.1 vs. 0.46 mm, before and after, respectively). It is unknown why these increases are so significant for membranes functionalised using diazonium salt **5** compared to **6**–**8** (vide infra), but regardless, these changes in physical properties are impressive from a simple and cost effective procedure. Carrying this same experiment out with samples functionalised using diazonium salts **6**–**8** gave a variety of results, with load before puncture increasing for the 3-(trfluoromethyl)benzenediazonium salt **6** by 55%, decreasing by 50% for the nitrile bearing salt **7**, and slightly decreasing for salt **8** (31%). However, in these latter cases, the changes to elongation before puncture were much less influenced by the exposure of the films to a humid environment, with all changes in elongation being less than 0.07 mm.

It should be noted that the membranes used in this analysis were cut from the same region of a larger silk membrane. This was done to minimise any variations in thickness that may affect these results. Additionally, the membranes were measured with callipers, with an accuracy of 10 µm, and no differences were found between (or within) each set of samples. Therefore, we attribute the changes in needle penetration to the treatments described above.

**Figure 9:**
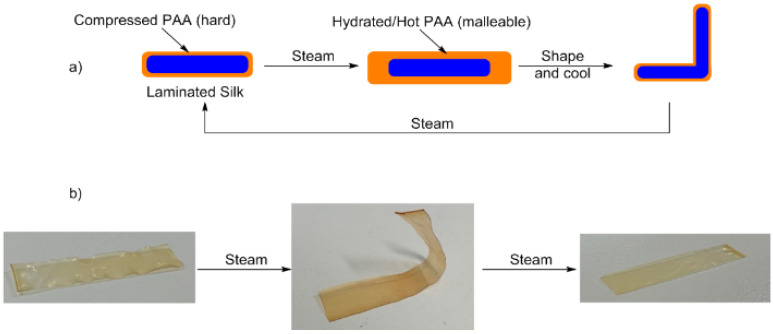
(**a**) Schematic of mouldable silk membranes and their reversible forming; (**b**) before and after moulding in steam for membranes functionalised using the 3-(trifluoromethyl)benzenediazonium salt **7** (larger image in ESI).

An unexpected result of exposing these films to a steam environment was the ability to manipulate these films into various shapes. It was noticed that when the films were steam treated, they became soft and malleable, similar to the untreated membranes, although upon cooling they would again become stiff. Thus, we were curious if these membranes, effectively possessing a laminated structure, could be formed into custom shapes for potential applications in personalised medicine. Recently, additive manufacturing of silk has been reported [36], and while this will undoubtedly serve as a source of membrane customization, using a method such as this will obviate laborious formulation into printable inks. Carrying out this process on all the samples showed the reversible formability, where the heated films were bent and allowed to cool (Figure 9b). Subsequent exposure to steam resulted in the membrane returning to its original form. The same treatment was conducted on an untreated sample, which did show some propensity for retention of the shape, although a significant amount of creep was observed (refer to ESI for image).

Attempts to carry this out using heat only (i.e., in the absence of moisture) immediately results in the membrane becoming crumpled and shrivelling. Thus, we believe that the ability to manipulate these laminated membranes into various shapes is the result of the polymer swelling with water, becoming malleable, and then evaporation of loosely bound water resulting in the ‘hardening’ of the surface tethered film. It is also important to note that the glass transition temperature (T_g_) of polyacrylic acid is ~106 °C [37]. This value is determined for the linear thermoplastic polymer of well-defined structure. The uncontrolled nature of this grafting process may result in polymeric materials on the surface which possess a slightly lower T_g_ and thus the remodelling of the polymer structure on the surface in addition to hydration may be responsible for these observations and the changes in physical properties before and after steam treatment.

## 4. Conclusions

Herein, we have shown a chemical means to generate surface-modified silk membranes using an inexpensive reductant (ascorbic acid). The electronics on the aryldiazonium salt initiator have a distinctive effect on the surface structure and polarity generated using this methodology. These effects are currently being examined in our laboratory using electrochemical reduction on controlled surfaces, and will be reported in due course. The treatments outlined above imbue the membranes with a variety of novel properties which have potential applications in preventing needle puncture and reversible formability.

## Supplementary Materials

The following are available online at https://www.mdpi.com/1996-1944/13/10/2252/s1, Figure S1: Untreated Membrane, Figure S2: Control membrane, Figure S3: 4-Nitrobenzene tetrafluoroborate treated membrane, Figure S4: 4-Cyanobenzene tetrafluoroborate treated membrane, Figure S5: 4-Ethynylbenzene tetrafluoroborate treated membrane, Figure S6: 3-(Trifluoromethyl)benzene tetrafluoroborate treated membrane, Figure S7: Untreated Membrane, Figure S8: Control membrane, Figure S9: 4-Nitrobenzene tetrafluoroborate treated membrane, Figure S10: 4-Cyanobenzene tetrafluoroborate treated membrane, Figure S11: 4-Ethynylbenzene tetrafluoroborate treated membrane, Figure S12: 3-(Trifluoromethyl)benzene tetrafluoroborate treated membrane, Figure S13: Untreated Membrane, Figure S14: Control membrane, Figure S15: 4-Nitrobenzene tetrafluoroborate treated membrane, Figure S16: 4-Cyanobenzene tetrafluoroborate treated membrane, Figure S17: 4-Ethynylbenzene tetrafluoroborate treated membrane, Figure S18: 3-(Trifluoromethyl)benzene tetrafluoroborate treated membrane, Figure S19: Schematic for Needle puncture apparatus, S20: Untreated Membrane, Figure S21 Control membrane, Figure S22: 4-Nitrobenzene tetrafluoroborate treated membrane, Figure S23: 4-Cyanobenzene tetrafluoroborate treated membrane, Figure S24: 4-Ethynylbenzene tetrafluoroborate treated membrane, Figure S25: 3-(Trifluoromethyl)benzene tetrafluoroborate treated membrane, Figure S26. (a) Untreated membrane; (b) Control Membrane; (c) 4-Nitrobenzene tetrafluoroborate treated membrane; (d) 4-Cyanobenzene tetrafluoroborate treated membrane; (e) 4-Ethynylbenzene tetrafluoroborate treated membrane; (f) 3-(Trifluoromethyl)benzene tetrafluoroborate treated membrane, Figure S27. (a) Schematic of the proposed mechanism of malleability; (b) Photos corresponding to each phase in the schematic.
