# Supplementary material for: Using In Situ Polymerization to Increase Puncture Resistance and Induce Reversible Formability in Silk Membranes
Source: Materials (Basel). 2020 May 14;13(10):2252. doi: 10.3390/ma13102252 (PMC7287606; doi:10.3390/ma13102252)
Supplement: Supplementary file 1 [file materials-13-02252-s001.pdf]

materials

MDPI

Supplementary Materials

Using *in Situ* Polymerization to Increase Puncture Resistance and Induce Reversible Formability in Silk Membranes

Nicholas S. Emonson, Daniel J. Eyckens, Benjamin J. Allardyce, Andreas Hendlmeier, Melissa K. Stanfield, Lachlan C. Soulsby, Filip Stojcevski* and Luke C. Henderson *

Carbon Nexus, Institute for Frontier Materials, Deakin University, Waurn Ponds, Victoria 3216, Australia; nemonson@deakin.edu.au (N.S.E.); dan.eyckens@deakin.edu.au (D.J.E.); ben.allardyce@deakin.edu.au (B.J.A.); ajhendlm@deakin.edu.au (A.H.); mstanfie@deakin.edu.au (M.K.S.); l.soulsby@deakin.edu.au (L.C.S.)

* Correspondence: f.stojcevski@deakin.edu.au (F.S.); luke.henderson@deakin.edu.au (L.C.H.)

Received: 24 April 2020; Accepted: 11 May 2020; Published: date

FTIR Spectra for all samples

AFM Images of all samples

Puncture testing of silk membranes

Images of Contact Angle for each Silk Membrane treatment

Reflectance Spectroscopy

Figure 9 from Manuscript (Enlarged)

FTIR Spectra for all samples.

All spectra are overlaid from one sample with each spectrum acquired from a different location on the silk membrane.

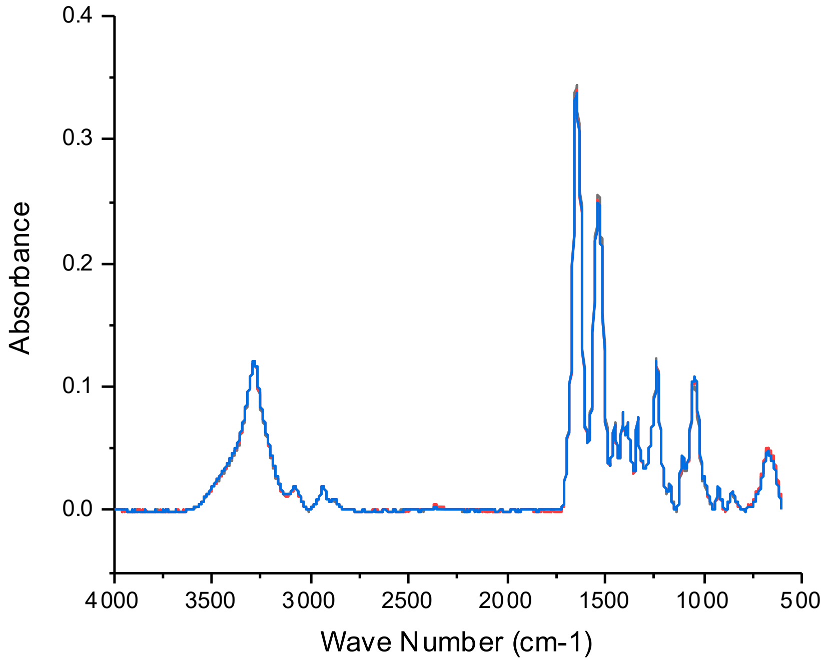

Figure S1. Untreated membrane.

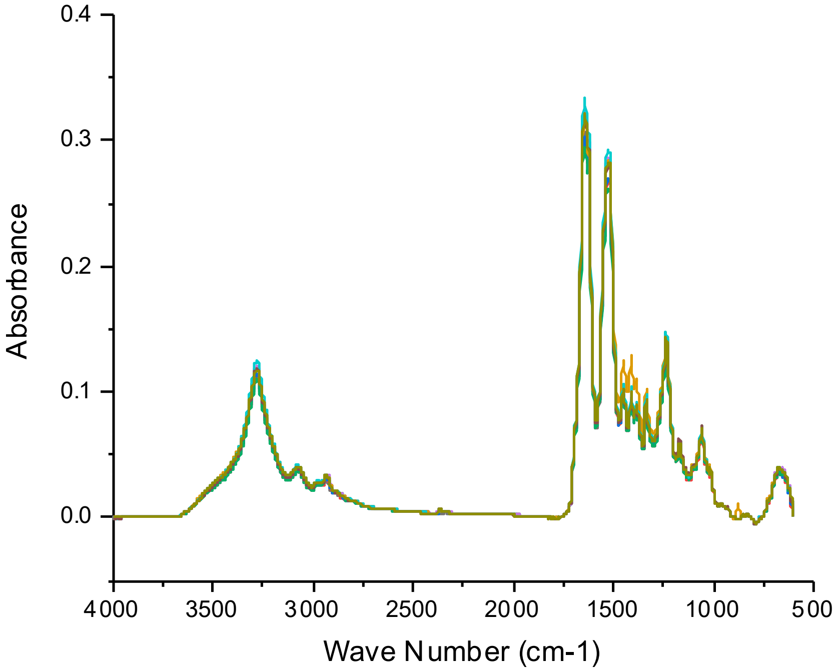

Figure S2. Control membrane.

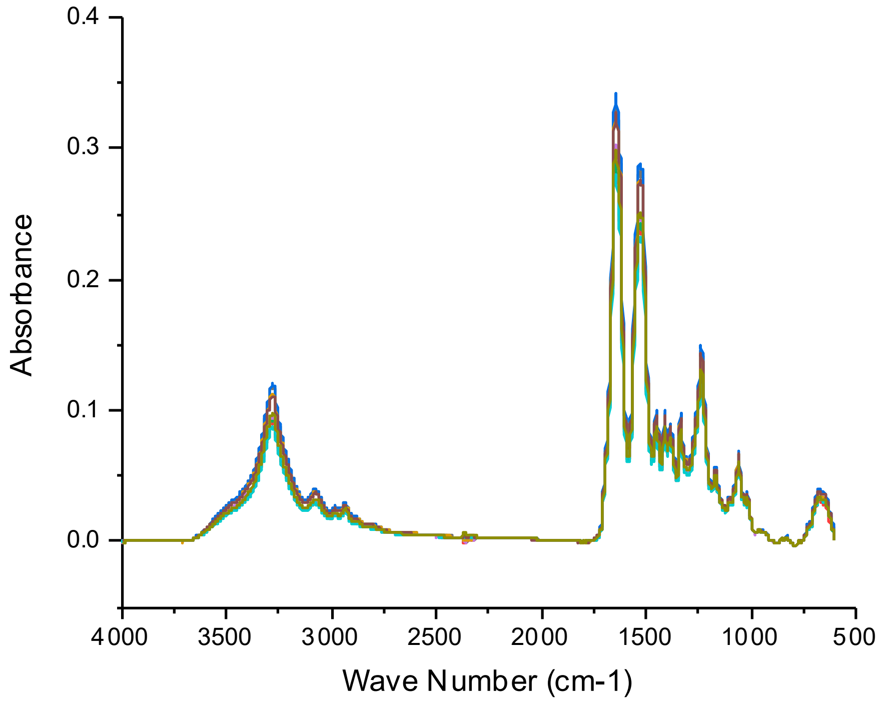

Figure S3. 4-Nitrobenzene tetrafluoroborate treated membrane.

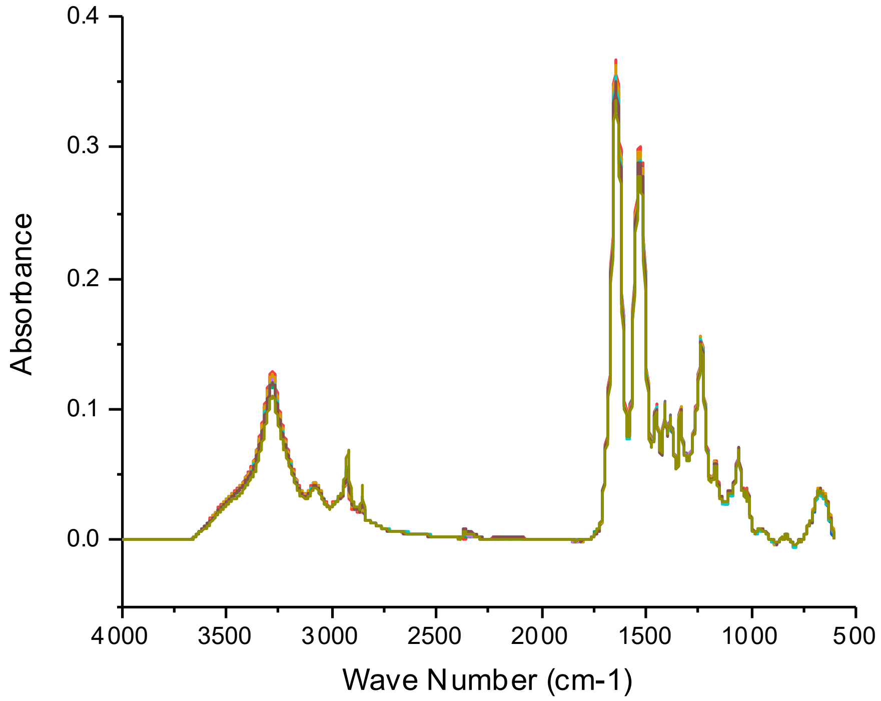

Figure S4. 4-Cyanobenzene tetrafluoroborate treated membrane.

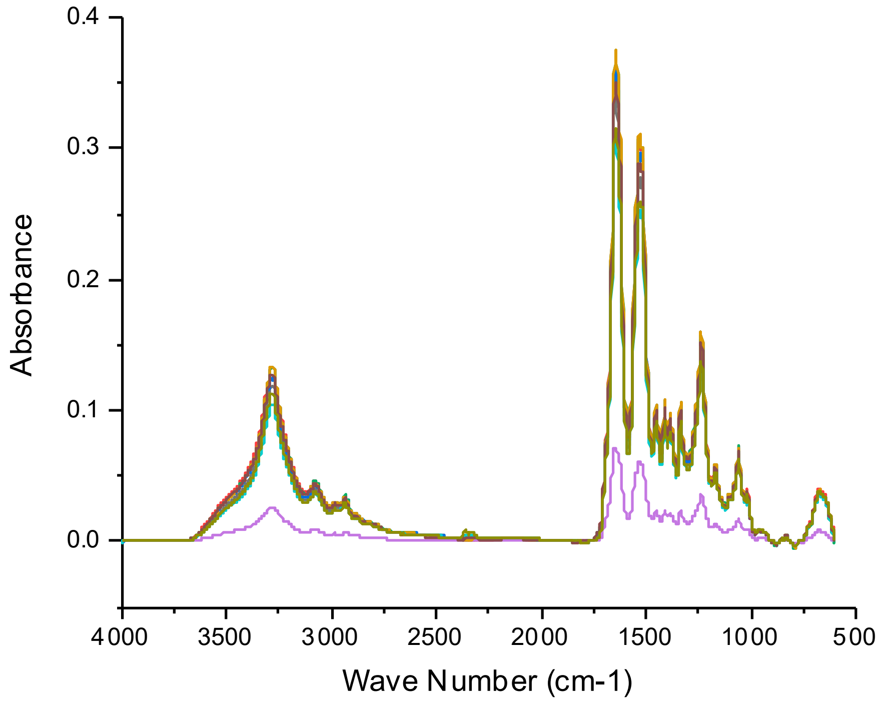

Figure S5. 4-Ethynylbenzene tetrafluoroborate treated membrane.

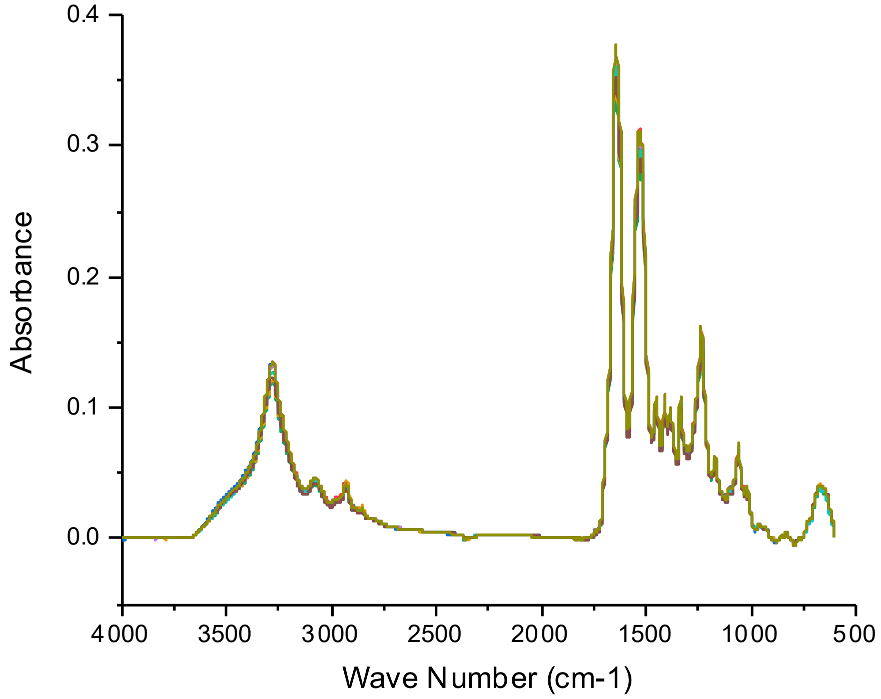

Figure S6. 3-(Trifluoromethyl)benzene tetrafluoroborate treated membrane.

AFM Images of all samples

All images are obtained from a 50 nm × 50 nm section of silk membrane.

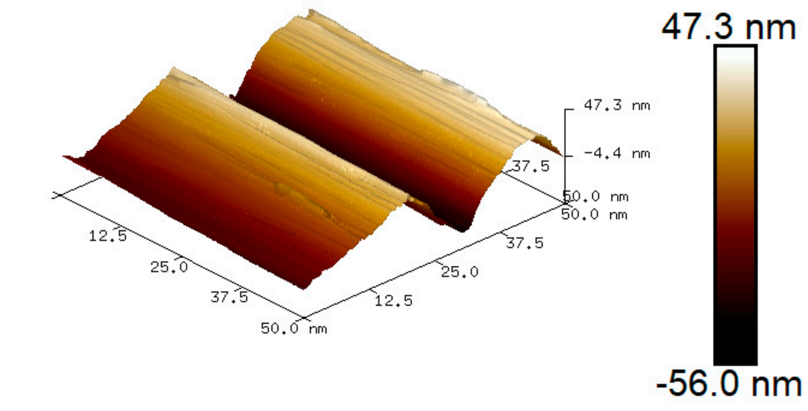

Figure S7. Untreated membrane.

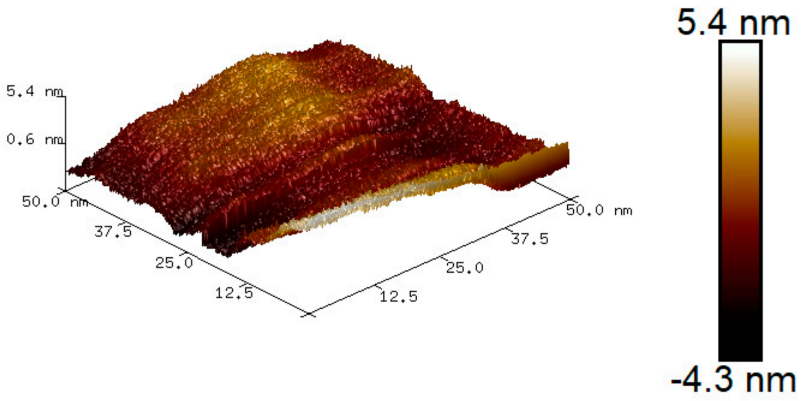

Figure S8. Control Membrane.

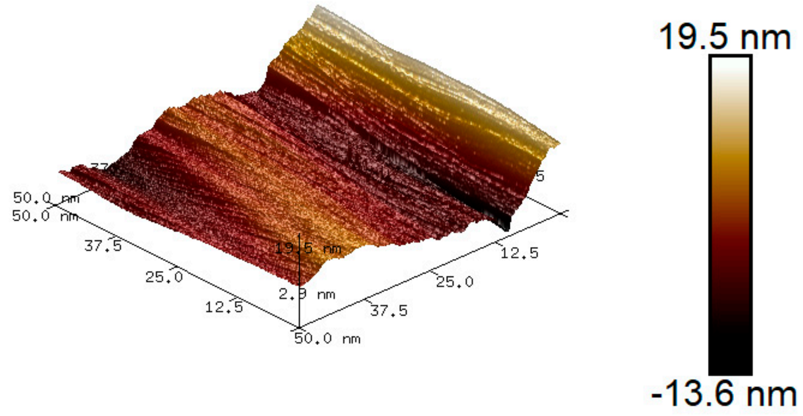

Figure S9. 4-Nitrobenzene tetrafluoroborate treated membrane.

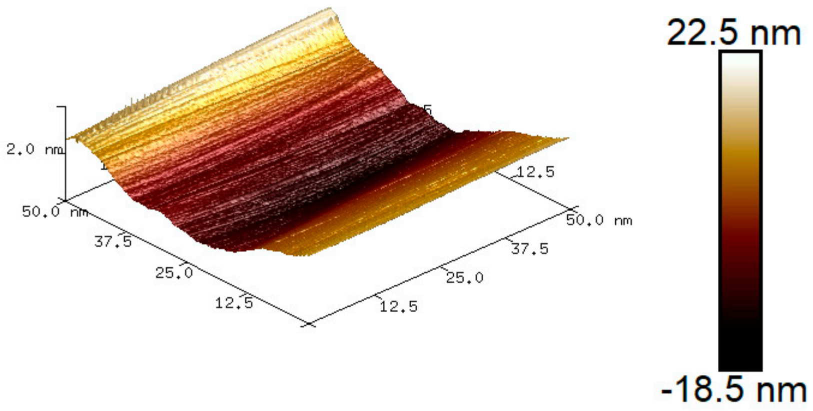

Figure S10. 4-Cyanobenzene tetrafluoroborate treated membrane.

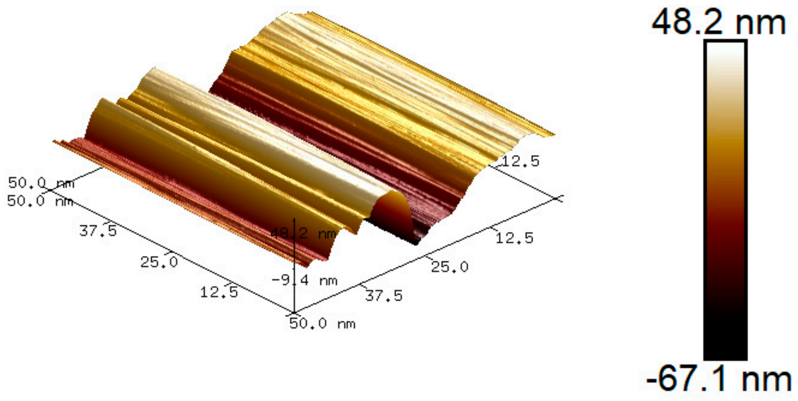

Figure S11. 4-Ethynylbenzene tetrafluoroborate treated membrane.

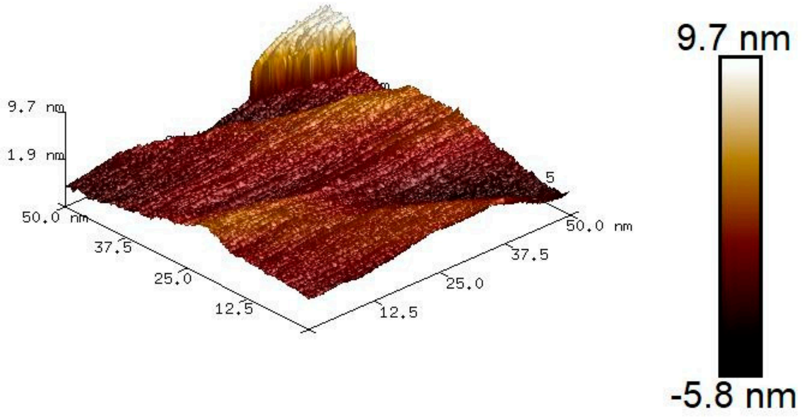

Figure S12. 3-(Trifluoromethyl)benzene tetrafluoroborate treated membrane.

Puncture testing of silk membranes

All images are obtained from a 50 nm × 50 nm section of silk membrane.

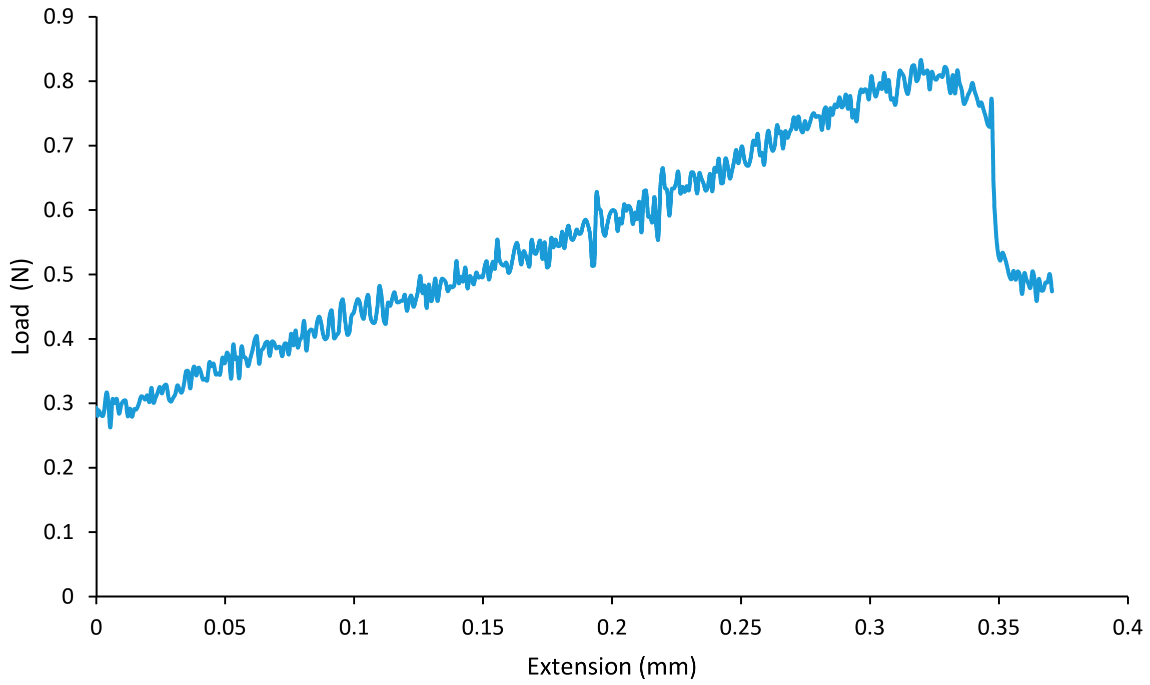

Figure S13. Untreated membrane.

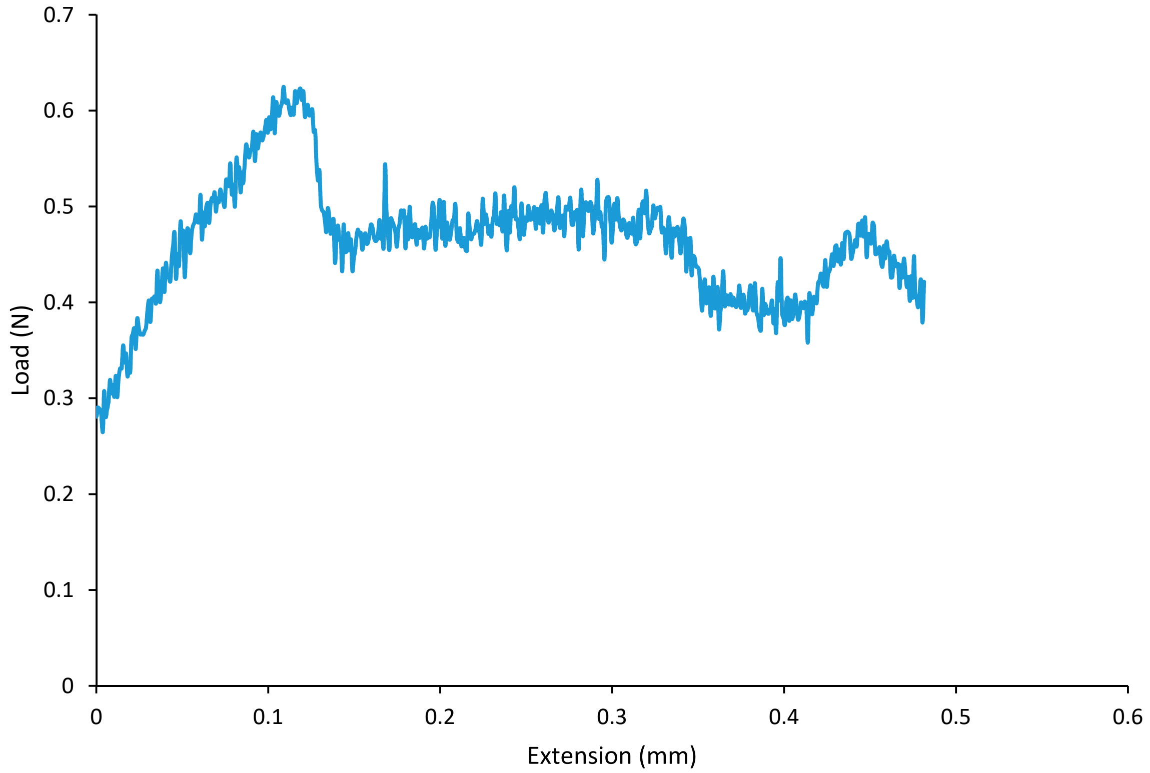

Figure S14. Control membrane.

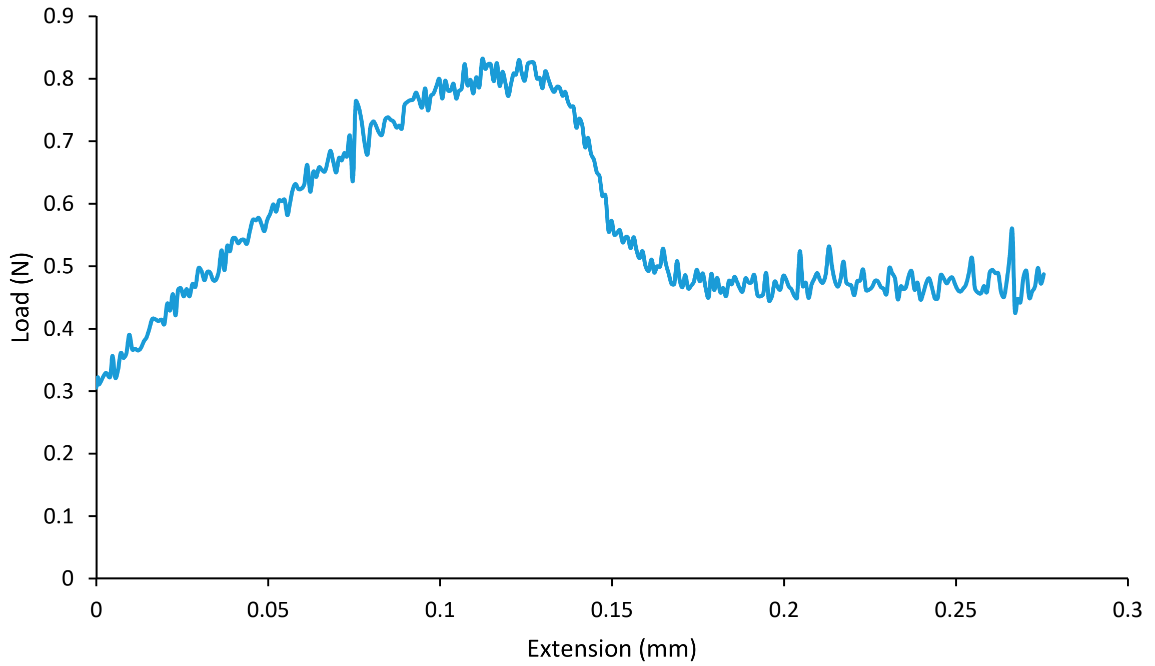

Figure S15. 4-Nitrobenzene tetrafluoroborate treated membrane.

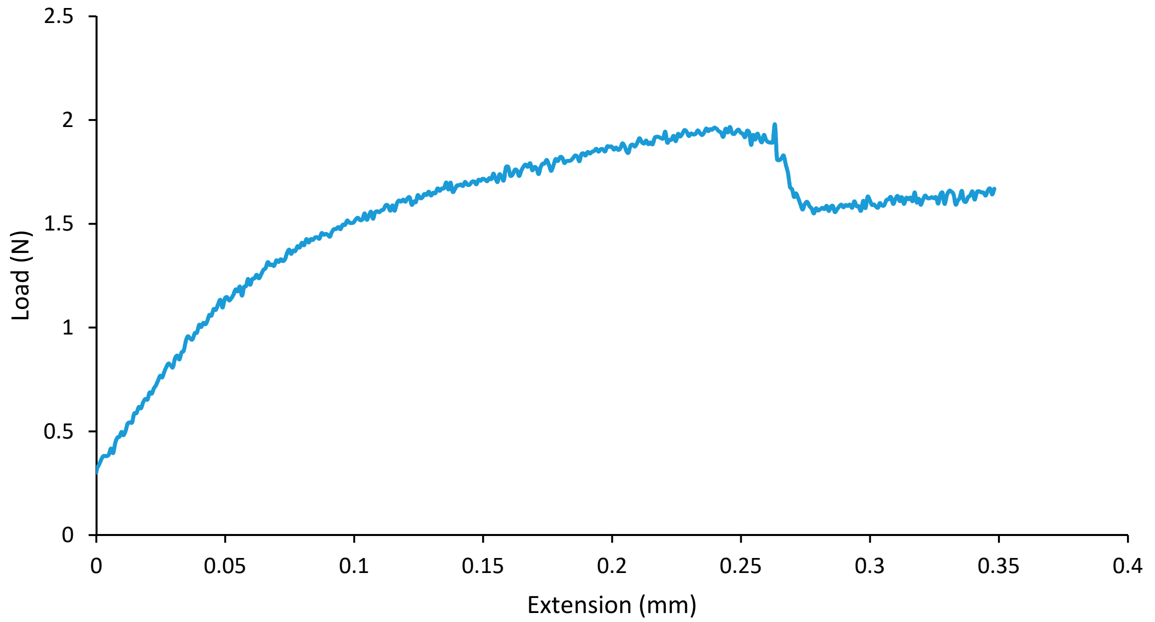

Figure S16. 4-Cyanobenzene tetrafluoroborate treated membrane.

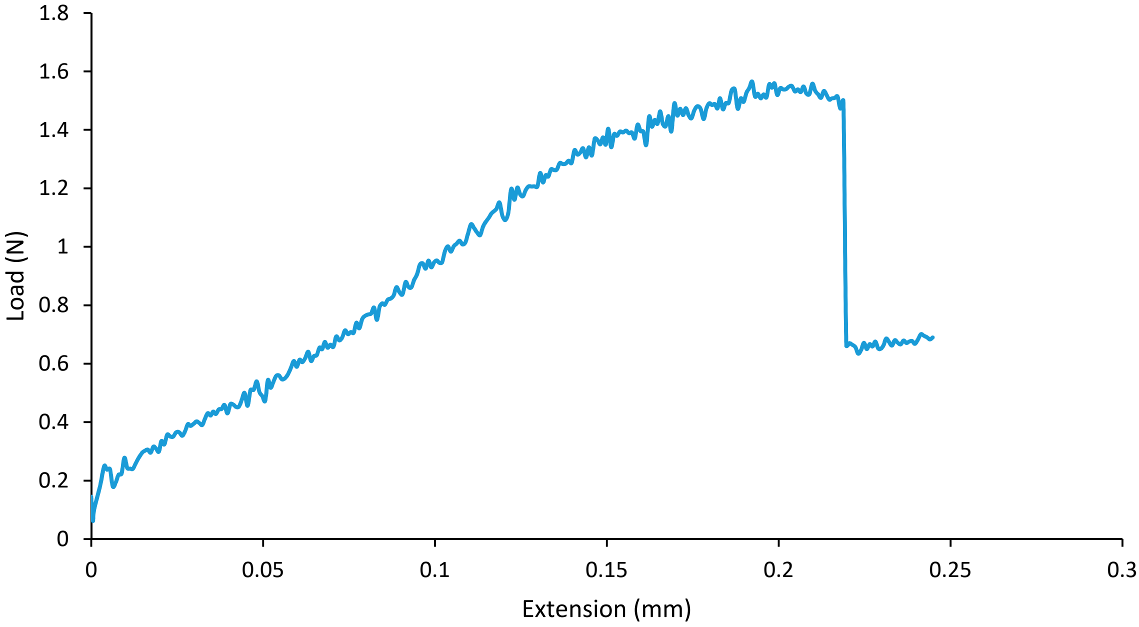

Figure S17. 4-Ethynlbenzene tetrafluoroborate treated membrane.

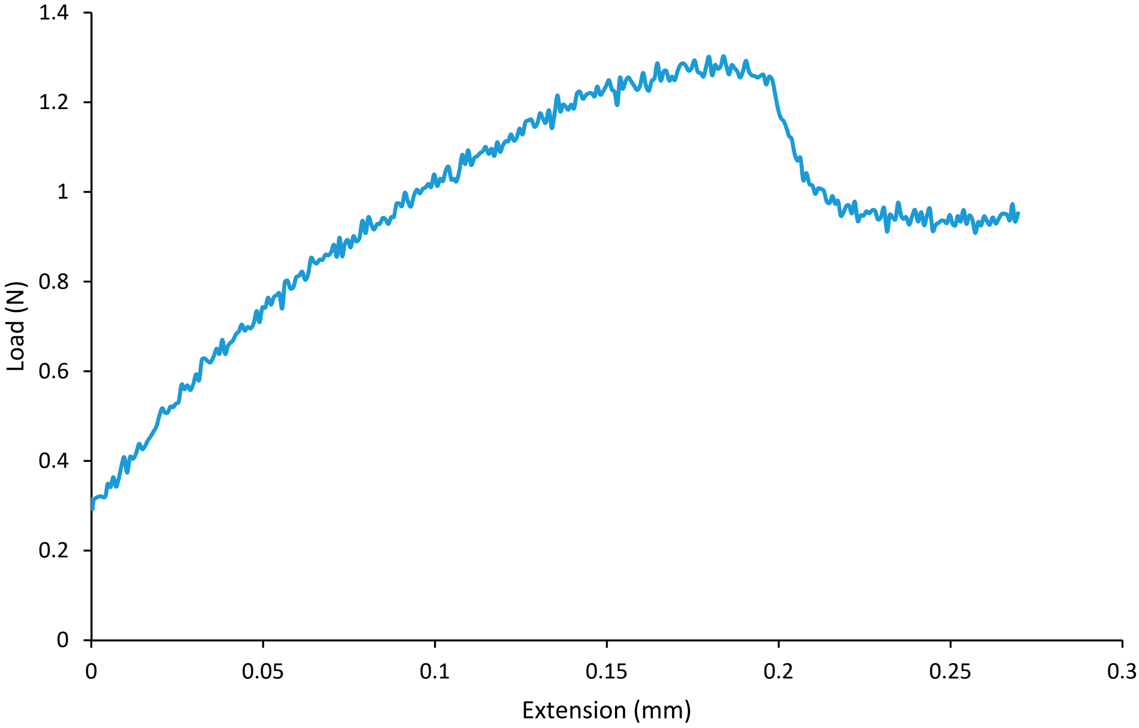

Figure S18. 3-(Trifluoromethyl)benzene tetrafluoroborate treated membrane.

Scheme of needle puncture test

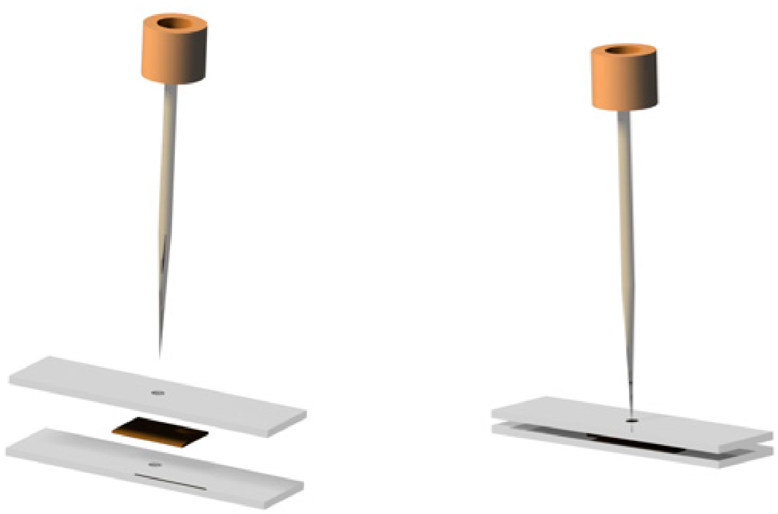

Figure S19. Schematic for Needle puncture apparatus.

Images of Contact Angle for each Silk Membrane treatment

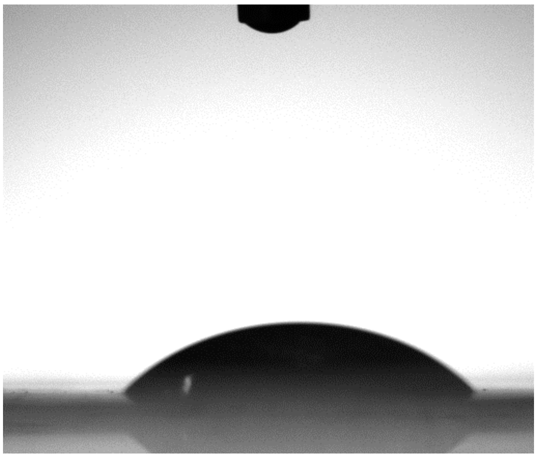

Figure S20. Untreated Membrane.

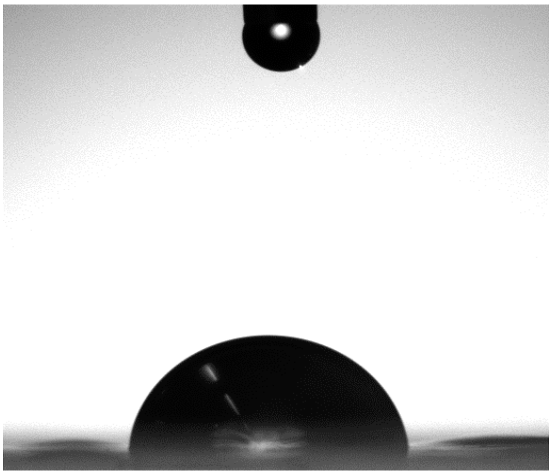

Figure S21. Control Membrane.

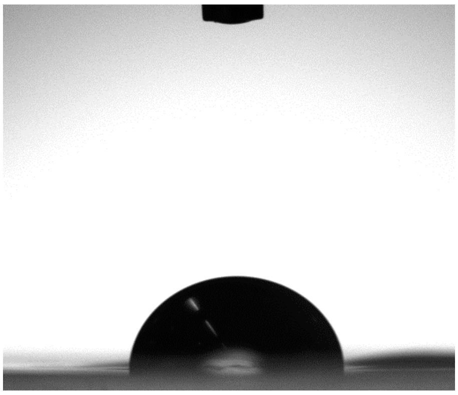

Figure S22. 4-Nitrobenzene tetrafluoroborate treated membrane.

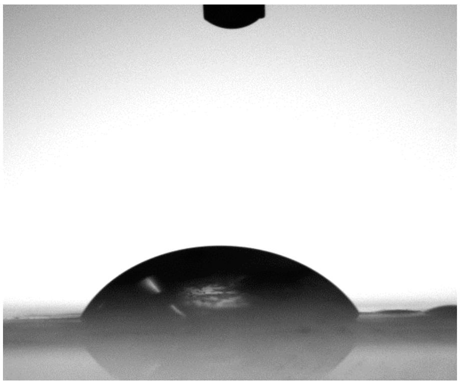

Figure S23. 4-Cyanobenzene tetrafluoroborate treated membrane

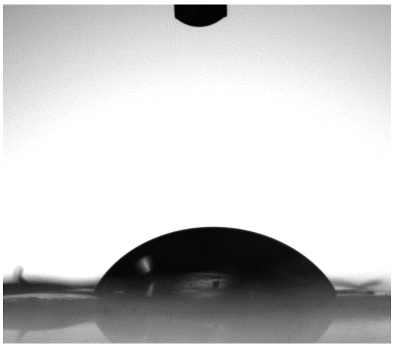

Figure S24. 4-Ethynylbenzene tetrafluoroborate treated membrane.

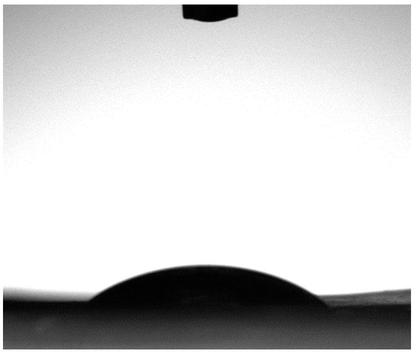

Figure S25. 3-(Trifluoromethyl)benzene tetrafluoroborate treated membrane.

Reflectance Spectroscopy

Reflection measurements were conducted with an Ocean Optics STS-VIS Miniature Spectrometer using SpectraSuite software. An Ocean Optics QR400-7-VIS-NIR 2 m reflection probe was coupled with an Ocean Optics HL-2000-FHSA tungsten halogen light source and the spectrometer to complete the reflection setup. A reflection probe holder was used to position the probe at an angel of 90°, with the face of probe placed approximately 3 mm from the surface of the silk samples. Once the reflection measurements were conducted, the spectra were then exported to Excel for graphing.

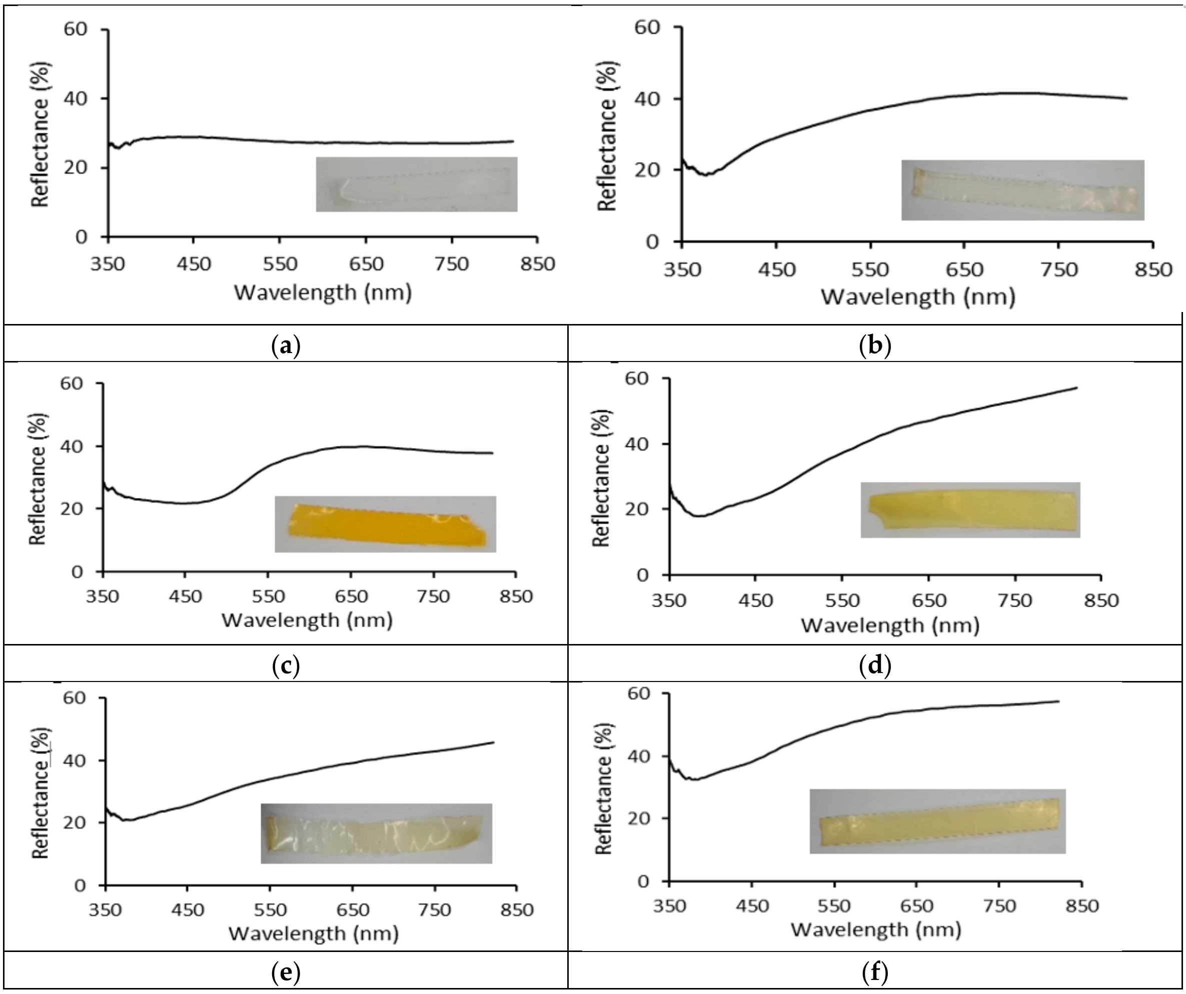

Figure S26. (**a**) Untreated membrane; (**b**) Control Membrane; (**c**) 4-Nitrobenzene tetrafluoroborate treated membrane; (**d**) 4-Cyanobenzene tetrafluoroborate treated membrane; (**e**) 4-Ethynylbenzene tetrafluoroborate treated membrane; (**f**) 3-(Trifluoromethyl)benzene tetrafluoroborate treated membrane.

Figure 9 from Manuscript (Enlarged)

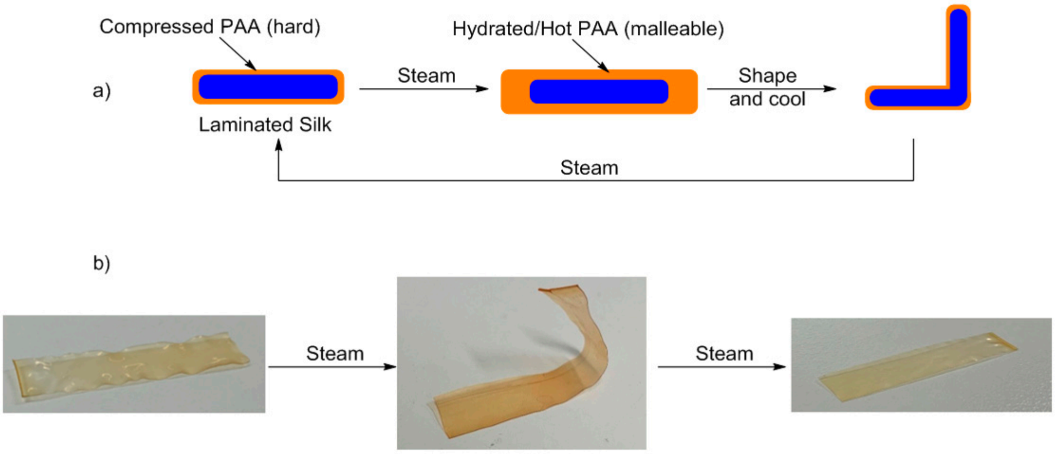

Figure S27. (**a**) Schematic of the proposed mechanism of malleability; (**b**) Photos corresponding to each phase in the schematic.

© 2020 by the authors. Submitted for possible open access publication under the terms and conditions of the Creative Commons Attribution (CC BY) license (http://creativecommons.org/licenses/by/4.0/).
